# The Triglyceride Glucose–Conicity Index as a Novel Predictor for Stroke Risk: A Nationwide Prospective Cohort Study

**DOI:** 10.3390/jcm14197086

**Published:** 2025-10-07

**Authors:** Xiaoqi Ye, Yan Li, Yebei Liang, Lihong Chen, Xingwu Ran

**Affiliations:** 1 Department of Endocrinology and Metabolism, West China Hospital, Sichuan University, Chengdu 610041, China; yexiaoqi@wchscu.edu.cn (X.Y.); yan_lee1993@163.com (Y.L.); chenlihong@scu.edu.cn (L.C.); 2 Innovation Research Center for Diabetic Foot, Diabetic Foot Care Center, West China Hospital, Sichuan University, Chengdu 610041, China; 3 Department of Geriatrics, The First Affiliated Hospital of Fujian Medical University, Fuzhou 350004, China

**Keywords:** triglyceride–glucose index, conicity index, triglyceride glucose–conicity index, stroke, prospective cohort study

## Abstract

**Background/Objectives:** The triglyceride–glucose index (TyG) and conicity index (CI) are markers of insulin resistance and abdominal obesity, respectively. However, their joint impact on stroke remains unclear. This study aimed to assess the association between the novel composite TyG–conicity index (TyG-CI = TyG × CI) and stroke risk. **Methods:** This prospective cohort study enrolled 8011 participants aged 45 years or older with no history of stroke at baseline, from the China Health and Retirement Longitudinal Study. Cox proportional hazards regression models were used to estimate the impact of TyG-CI on the risk of incident stroke. Restricted cubic spline regressions were applied to estimate possible nonlinear associations. The predictive performance was evaluated using the area under the receiver operating characteristic curve (AUC) and time-dependent Harrell ‘s C-index. **Results:** Over a mean follow-up of 8.36 years, 753 participants (9.4%) experienced stroke. After adjusting for potential confounders, compared with the lowest quartile, the adjusted HRs of incident stroke for participants with the highest quartile of TyG, CI, and TyG-CI were 1.36 (95%CI 1.05–1.76), 1.43 (95%CI 1.15–1.78), and 1.69 (95%CI 1.31–2.17), respectively. Restricted cubic spline analysis confirmed a linear relationship between TyG-CI and stroke risk (*p* for nonlinearity = 0.90). The AUC was highest for TyG-CI (0.594), exceeding TyG (0.575) and CI (0.575) with statistically significant differences (all *p* < 0.05). **Conclusions:** Elevated TyG-CI indicates a higher stroke risk, with superior predictive value compared to its components alone, providing a promising indicator that is simple, reliable, and inexpensive for identifying stroke risk in clinical practice.

## 1. Introduction

According to the most recent Global Burden of Disease study, stroke ranks as the second leading cause of death and the third leading cause of disability-adjusted life-years [1]. Globally, the estimated direct and indirect costs associated with stroke exceed US$891 billion annually [2]. In China, stroke remains the foremost cause of mortality and disability, accounting for approximately one-third of worldwide stroke-related deaths, with its prevalence steadily rising [3,4]. Given that stroke is highly preventable, identifying modifiable risk factors is crucial.

Metabolic risk factors play a predominant role in stroke occurrence, contributing to nearly 70% of all strokes [5]. Abdominal obesity (AO), a key metabolic risk factor, substantially elevates the risk for cardiovascular disease (CVD). Conicity index (CI), an anthropometric measure of AO that incorporates waist circumference (WC), height, and weight to assess fat distribution, is considered a superior indicator of AO than WC, waist-to-height ratio (WtHR), and body mass index (BMI) [6]. CI is associated with hypertriglyceridemia, decreased high-density lipoprotein cholesterol (HDL-C), and elevated low-density lipoprotein cholesterol (LDL-C), all of which heighten cardiovascular risk [7]. Insulin resistance (IR), a well-established risk factor for type 2 diabetes and CVD, is conventionally determined by high insulin-normoglycemic clamp and homeostatic model assessment for insulin resistance (HOMA-IR) [8,9,10]. Triglyceride–glucose index (TyG), a new and reliable index for assessing IR derived from fasting plasma glucose (FPG) and triglycerides (TG), offers a more straightforward, cost-effective, and stable alternative to conventional methods [11,12]. Several studies have shown that TyG is a promising biomarker for different diseases, especially stroke, where TyG is strongly associated with stroke incidence and may even be superior to HOMA-IR in predicting stroke risk [13,14,15].

Emerging evidence has highlighted the improved predictive properties of composite metabolic indices for stroke risk, with even greater efficacy observed when these indices are used in combination [16,17,18]. AO and IR are usually concomitant. Therefore, we developed the triglyceride glucose–conicity index (TyG-CI), integrating TyG and CI, to provide a comprehensive assessment of systemic IR and visceral adiposity. In this study, based on the China Health and Retirement Longitudinal Study (CHARLS), we investigated the relationship between TyG-CI and stroke risk and further evaluated whether this novel composite index better predicts incident stroke.

## 2. Materials and Methods

### 2.1. Study Design and Population

This prospective cohort study utilized data from the CHARLS, an ongoing nationally representative longitudinal survey examining the economic, social, and health conditions of Chinese adults aged 45 years and older. The CHARLS samples were chosen through a multi-stage, probability-proportional-to-size sampling strategy to ensure national representativeness. The national baseline survey was conducted between June 2011 and March 2012, enrolling a total of 17,708 participants from 28 provinces, 150 county-level districts, and 450 villages in China. All participants were interviewed using a standardized questionnaire to collect essential information. Follow-up surveys were conducted every two years to track changes in participants’ health status. Detailed descriptions of the CHARLS study design, sampling methodology, and data collection procedures have been published previously [19]. The present study utilized the baseline survey (2011) data to assess exposures and covariates. Participants were then followed for incident stroke events through the subsequent waves (2013, 2015, 2018, and 2020).

**Figure 1:**
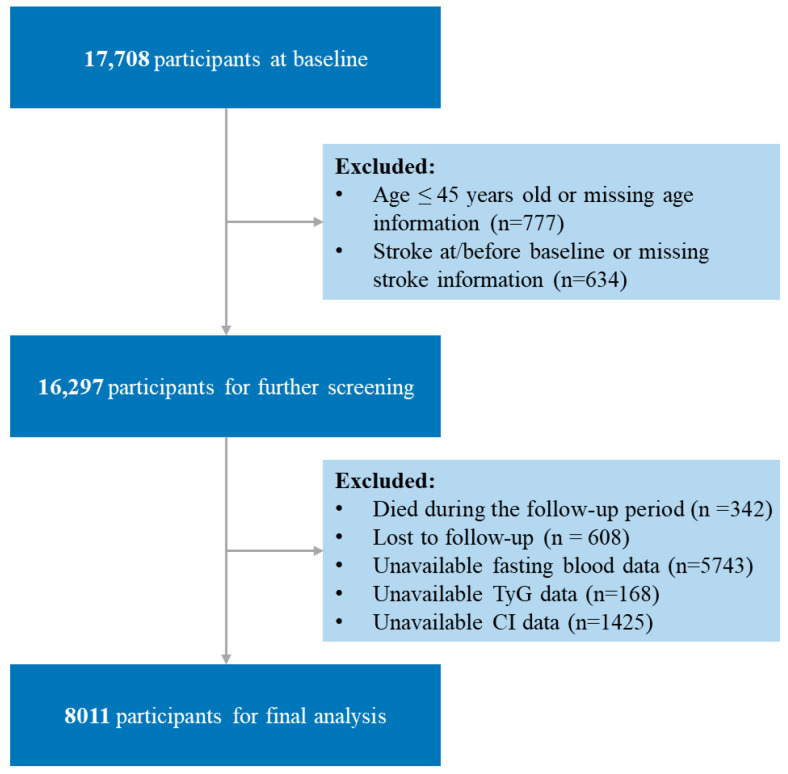
Flow chart of the study population. TyG, triglyceride–glucose; CI, conicity index; TyG-CI, triglyceride glucose–conicity index.

In brief, a total of 17,708 participants were enrolled in the baseline survey (2011). Then, for our analysis, 9697 participants were excluded based on the following criteria: age under 45 years or missing age information at baseline (*n* = 777); self-reported stroke diagnosis at/before baseline or missing stroke information (*n* = 634); died during the follow-up period (*n* = 342); lost to follow-up (*n* = 608); unavailable fasting blood data at baseline (*n* = 5743); unavailable TyG data at baseline (*n* = 168); unavailable CI data at baseline (*n* = 1425). Finally, a total of 8011 participants were retained in the final study. The participant selection process is detailed in Figure 1.

The CHARLS study was approved by the Institutional Review Board of Peking University (approval number: IRB00001052-11015 for the household survey and IRB00001052-11014 for blood samples). Written informed consent was obtained from all participants. This study adhered to the Strengthening the Reporting of Observational Studies in Epidemiology (STROBE) guidelines [20], and the STROBE checklist is shown in Table S1.

### 2.2. Assessment of Exposure

The exposures of interest in this study were the TyG, CI, and their product, the novel TyG-CI. These indices were calculated as follows:

TyG = ln[TG (mg/dL) × FPG (mg/dL)/2]

CI = WC (m) × (0.109^−1^) × ([Weight (kg)/Height (m)]^−0.5^)

TyG-CI = TyG × CI

All biomarker and anthropometric data used to calculate these indices were assessed at the baseline survey by trained staff following standardized protocols.

After an overnight fast of at least 8 h, venous blood samples were drawn from participants by trained nurses. The samples were immediately processed, frozen at −20 °C, and subsequently transported to a central laboratory at the Chinese Center for Disease Control and Prevention in Beijing for analysis. TG and FPG levels were measured using the enzyme colorimetric assays. Both within-run and between-run coefficients of variation for TG and FPG were less than 2%.

Body height and weight were measured with participants in light clothing and standing upright without shoes on the instruments. Weight was measured to the nearest 0.1 kg using a calibrated digital scale (Omron™ HN-286 scale, Kerer Technology Co., Ltd., Yangzhou, China). Height was measured to the nearest 0.1 cm using a portable stadiometer (Seca™ 213 stadiometer, Seca Co., Ltd., Hangzhou, China). WC was measured using the tape measure at the umbilical level with the participant maintaining a standing position.

### 2.3. Assessment of Outcome

The primary outcome of this study was stroke. In accordance with previous studies, we identified incident stroke through a questionnaire where individuals self-reported “yes” to the standardized question, “Have you been diagnosed with a stroke by a doctor?”. For stroke cases, the time-to-event was calculated from baseline to reported onset. For participants without a reported stroke, the follow-up duration spanned the baseline to the final survey.

### 2.4. Definitions of Covariates

At the baseline assessment, trained interviewers collected information on sociodemographic and health-related information. Sociodemographic data included age, sex, residence, marital status, and educational level. Health-related information included lifestyle factors (smoking and drinking status), anthropometric data (body weight, height, WC, systolic blood pressure [SBP] and diastolic blood pressure [DBP]), medical history (diabetes, hypertension, dyslipidemia, heart disease, and kidney disease) and its treatment, and laboratory test results (total cholesterol [TC], HDL-C, LDL-C, TG, FPG, glycosylated hemoglobin A1c [HbA1c], creatinine). TC, HDL-C, LDL-C, TG, and FPG were measured using enzymatic colorimetric assays. HbA1c was quantified by Boronate affinity HPLC. Serum creatinine was assessed using a Rate-blanked and compensated Jaffe creatinine method. The estimated glomerular filtration rate (eGFR) was calculated using the 2021 Chronic Kidney Disease Epidemiology Collaboration equation [21]. Diabetes was defined based on self-reported physician-diagnosed history of diabetes, FPG ≥ 126 mg/dL, HbA1c ≥ 6.5%, or current use of antidiabetic medication. Hypertension was defined based on self-reported physician-diagnosed history of hypertension, SBP ≥ 140 mmHg, DBP ≥ 90 mmHg, or current use of antihypertensive medication. Dyslipidemia was determined based on self-reported physician-diagnosed history of dyslipidemia, TC ≥ 240 mg/dL, TG ≥ 150 mg/dL, LDL-C ≥ 160 mg/dL, HDL-C < 40 mg/dL, or current use of lipid-lowering medication. Heart disease was defined based on self-reported physician-diagnosed history of heart disease (heart attack, coronary heart disease, angina, congestive heart failure, or other heart problems). Kidney disease was defined based on self-reported physician-diagnosed history of kidney disease (except for tumor or cancer) or eGFR < 60 mL/min/1.73 m^2^.

### 2.5. Statistical Analysis

Baseline indicators were categorized by TyG-CI quartiles and stroke status. Continuous variables were presented as median (Quartile 1 [Q1], Quartile 3 [Q3]), and categorical variables were presented as numbers (proportions). Baseline differences across groups were compared using the Kruskal-Wallis test for continuous variables and Pearson’s chi-squared test for categorical variables, as appropriate. Missing data (≤2% for most variables, except alcohol consumption [5.14%]) were imputed using the R package (version 4.4.0) “missForest”, a nonparametric missing value imputation for mixed-type data utilizing the random forest algorithm [22].

Cox proportional hazards regression models were employed to estimate the hazard ratios (HRs) and 95% confidence intervals (CIs) for stroke risk associated with TyG, CI, and TyG-CI. Three models were used: Model 1 (not adjusted for other covariates), Model 2 (adjusted for age, sex, residence, marital status, educational level, smoking status, and drinking status), and Model 3 (adjusted for the variables in Model 2 plus BMI, hypertension, dyslipidemia, diabetes, heart disease, and kidney disease. Potential nonlinear association between TyG-CI and stroke risk was estimated using 3-knotted restricted cubic spline (RCS) regression, adjusting for covariates consistent with model 3.

The predictive performance of TyG, CI, and TyG-CI for stroke incidence was evaluated using the area under the curve (AUC) from receiver operating characteristic (ROC) curve analysis. To statistically compare the differences in AUC values, we employed DeLong’s test for two correlated ROC curves. In addition, the time-dependent Harrell’s C-index was also computed to evaluate the predictive accuracy of TyG, CI, and TyG-CI for stroke incidence over time.

Subgroup analyses (by age, sex, smoking status, drinking status, hypertension, dyslipidemia, diabetes, heart disease, and kidney disease status) were performed using the Cox regression model. Potential interactions between TyG-CI and the other covariates on incident stroke were tested using the Wald test by introducing a product variable to the regression models. Sensitivity analyses were also conducted to evaluate the robustness and reliability of our findings, including the use of complete case analysis without imputation of missing covariates and excluding participants receiving antihyperglycemic treatment, antihypertensive treatment, or lipid-lowering treatment.

All analyses were conducted with R version 4.0.2 (R Foundation for Statistical Computing). A two-sided *p* value <0.05 was considered statistically significant.

## 3. Results

### 3.1. Baseline Characteristics of Study Participants

The study cohort comprised 8011 participants, with a median age of 58 years and 46% males. The baseline characteristics of study participants according to TyG-CI quartiles are summarized in Table 1, and those based on stroke status are presented in Table S2. At baseline, the median of TyG was 8.6 (8.2, 9.0), the median of CI was 1.3 (1.2, 1.3), and the median of TyG-CI was 11.0 (10.2, 11.9). Statistically significant differences were observed in almost all baseline characteristics among the four TyG-CI quartile subgroups, except for education level and kidney disease. Participants with higher TyG-CI also had higher BMI, SBP, DBP, TC, TG, LDL-C, FPG, and HbA1c, but lower HDL-C and eGFR. There were no significant differences in baseline characteristics between the participants with complete data and those with imputed data (Table S3).

### 3.2. Association of TyG, CI, and TyG-CI with Stroke Risk

**Figure 2:**
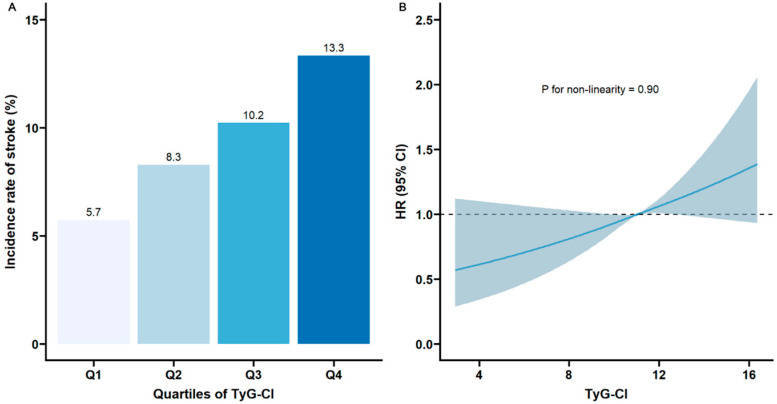
Nonlinear associations of TyG-CI with stroke risk: (**A**) The incidence of stroke across different quartiles of TyG-CI; (**B**) restricted cubic spline regression analysis of TyG-CI with stroke risk (the solid line represents the regression curve, and the shaded area represents the 95% confidence interval). TyG, triglyceride–glucose; CI, conicity index; TyG-CI, triglyceride glucose–conicity index.

Over a mean follow-up period of 8.36 years, 753 participants experienced stroke, resulting in an incidence rate of 9.4%. Table 2 shows the association of TyG, CI, and TyG-CI with stroke risk. Participants were divided into quartiles based on their rank according to TyG, CI, and TyG-CI values, ensuring an equal distribution of participants across groups wherever possible. The approximate values corresponding to these quartile boundaries were as follows: for TyG, Q1: ≤8.22, Q2: 8.22–8.59, Q3: 8.59–9.02, Q4: >9.02; for CI, Q1: ≤1.23, Q2: 1.23–1.28, Q3: 1.28–1.34, Q4: >1.34; for TyG-CI, Q1: ≤10.25, Q2: 10.25–11.04, Q3: 11.04–11.92, Q4: >11.92. After adjusting for potential confounders (Model 3), compared with the lowest quartile (Q1), the adjusted HRs of incident stroke for participants with the highest quartile of TyG, CI, and TyG-CI were 1.36 (95%CI 1.05–1.76), 1.43 (95%CI 1.15–1.78), and 1.69 (95%CI 1.31–2.17), respectively. Figure 2A illustrates the stroke incidence rate categorized by TyG-CI quartiles, which steadily rises with increasing TyG-CI values from 5.7% (Q1) to 13.3% (Q4). RCS analysis indicated a linear association between TyG-CI and stroke risk (*p* for nonlinearity = 0.90, Figure 2B).

### 3.3. Predictive Value of TyG-CI in Incident Stroke

**Figure 3:**
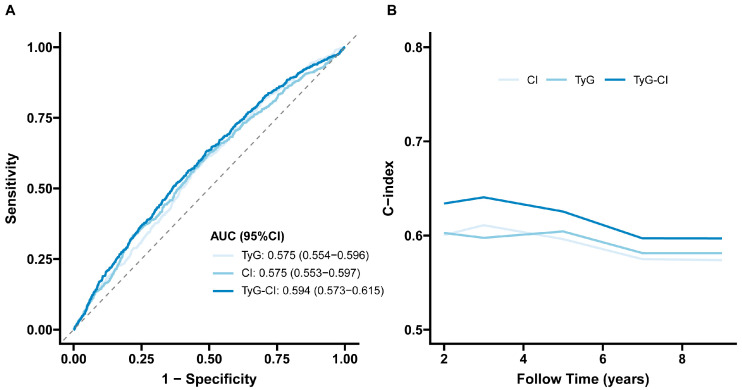
Predictive performance of the TyG, CI, and TyG-CI on the stroke risk: (**A**) receiver operating characteristic curve; (**B**) time-dependent Harrell’s C-index. TyG, triglyceride–glucose; CI, conicity index; TyG-CI, triglyceride glucose–conicity index. AUC, Area under the curve; 95%CI, 95% confidence interval.

The ROC curve indicated that the predictive performance of TyG-CI for incident stroke was superior to TyG and CI alone. The AUC for TyG-CI was 0.594 (95%CI 0.573–0.615), higher than TyG (0.575, 95%CI 0.554–0.596) and CI (0.575, 95%CI 0.553–0.597) (Figure 3A), with significant differences between these AUCs (TyG-CI vs. TyG: *p* value = 0.02; TyG-CI vs. CI: *p* value = 0.01). Meanwhile, time-dependent Harrell’s C-index further revealed that TyG-CI exhibited the highest predictive capability for risk of incident stroke compared with TyG and CI alone (Figure 3B).

### 3.4. Subgroup and Sensitivity Analyses

The associations between TyG-CI and incident stroke among different subgroups are shown in Table S4. After adjusting for potential confounders, no significant interactions were found between TyG-CI and subgroup variables (all *p* for interactions >0.05). The results of the sensitivity analyses were largely consistent with the primary findings. First, the exclusion of participants receiving antihyperglycemic treatment, antihypertensive treatment, or lipid-lowering treatment did not lead to any material changes in the results. Additionally, the findings remained robust after performing complete data analyses, further supporting the reliability of the current conclusions (Table S5).

### 3.5. Risk Factors for Stroke in Individuals with Low TyG-CI Values

To further understand the risk factors for stroke in individuals with low TyG-CI values, we conducted a subgroup analysis of participants in the first quartile (Q1) of TyG-CI (*n* = 2003). Among them, 115 (5.7%) developed stroke. Compared to those without stroke, these individuals had a higher prevalence of hypertension, heart disease, and dyslipidemia, as well as elevated TG levels (Table S6). Multivariate Cox regression identified hypertension, heart disease, and elevated TG as independent risk factors for stroke in this subgroup (Table S7).

## 4. Discussion

In this nationwide prospective cohort study of 8011 Chinese adults aged ≥45 years, we investigated the association between TyG-CI, a novel composite index integrating IR and AO proposed for the first time in this study, and the risk of incident stroke. The results demonstrated that TyG-CI was an independent predictor of incident stroke, exhibiting stronger predictive capability than TyG or CI alone. Additionally, the relationship between TyG-CI and stroke risk showed a linear pattern. No significant interaction was identified between TyG-CI and subgroups on stroke risk. Sensitivity analyses consistently validated the primary findings.

TyG is recognized as a reliable, inexpensive, and simple surrogate for IR [12,23]. IR contributes to the development of stroke through multiple mechanisms, including endothelial dysfunction, platelet activation, heightened sympathetic nervous system activity, and impaired cardiac autonomic function [24,25,26,27]. Our study reiterates the well-established association between elevated TyG levels and increased stroke risk, aligning with previous research findings. A large-scale, community-based prospective cohort study showed that elevated baseline and long-term TyG levels are associated with an increased risk of stroke, especially ischemic stroke, in American adults [28]. Two meta-analyses confirmed that TyG is an independent risk factor for stroke, and elevated TyG confers a higher risk of stroke occurrence, recurrence, and mortality [13,29].

AO has long been acknowledged as a critical risk factor for cerebrovascular events. CI is determined as an indicator of body fat distribution, with a high ability to discriminate underlying AO [30]. Emerging evidence suggests that CI may have a significant impact on cardiovascular health. For example, a cross-sectional study reported that CI was significantly associated with IR, hypertension, and dyslipidemia [31]. Tonding SF et al. found CI was the body adiposity marker best associated with a high risk of fatal coronary heart disease through a cross-sectional study of 420 patients with type 2 diabetes [32]. In a cross-sectional study of 3199 participants, Motamed et al. established that CI outperformed traditional central obesity indices (such as WC, waist-to-height ratio, and abdominal volume index) in predicting 10-year cardiovascular risk [33]. This was corroborated by Chung et al., who reported CI had a more discriminatory accuracy for CVD risk compared with other obesity parameters [7]. Of note, a most recent prospective study found a positive association between CI and the risk of both CVD mortality and all-cause mortality, and the highest CI values had a higher risk of both CVD mortality (HR 1.51, 95% CI 1.16–1.97) and all-cause mortality (HR 1.58, 95% CI 1.38–1.82) compared with the other groups [34]. Despite these advances, the specific relationship between CI and stroke risk remains unexplored in prospective cohorts. Our study addresses this critical knowledge gap through longitudinal analysis, establishing for the first time that higher baseline CI levels were associated with an increased stroke risk.

Given the complementary roles of TyG (reflecting systemic IR) and CI (indicating AO) in stroke pathogenesis, we hypothesized that integrating these indices into TyG-CI would offer a more accurate prediction of stroke risk. Our results support this hypothesis: TyG-CI not only demonstrated a stronger association with stroke risk but also improved predictive accuracy over its individual components. The absence of significant subgroup interactions underscores the generalizability of these findings. Clinically, TyG-CI offers a pragmatic tool for identifying high-risk individuals, enabling targeted interventions to mitigate stroke burden.

The better predictive capacity of the TyG-CI composite index compared to its individual components (TyG or CI) may stem from its synergistic integration of dual metabolic pathways: TyG primarily reflects systemic IR, and CI captures central obesity-related fat distribution. Both conditions are well-established drivers of atherosclerotic pathogenesis. Notably, previous studies have shown that TyG and CI are significantly associated with carotid artery intima-media thickness and carotid plaque assessed by carotid ultrasound [35,36]. This provides a biological plausibility for why TyG-CI serves as an excellent marker for elevated stroke risk, which is often the clinical consequence of atherosclerosis. In addition, there are individual variations in biomarker levels, with TyG and CI offering insights into IR and AO changes from different perspectives. By integrating TyG and CI, the TyG-CI composite index could avoid the intrinsic limitations of either index alone, such as the potential underestimation of metabolic risk in normal-weight individuals with visceral fat and elevated TyG. This combined approach enhances the ability to identify and stratify individuals at higher risk.

Furthermore, we observed that even among participants with low TyG-CI values, traditional risk factors such as hypertension, heart disease, and hypertriglyceridemia were significantly associated with stroke risk. This suggests that while TyG-CI is a robust composite metabolic marker for stroke, it does not fully capture all pathways leading to stroke, particularly those mediated by established cardiovascular comorbidities. Meanwhile, this also highlights that even in individuals with a favorable metabolic profile in terms of IR and AO, aggressive management of traditional cardiovascular risk factors remains critically important for stroke prevention.

Despite providing valuable insights into the association between TyG-CI and stroke risk, several limitations in this study must also be considered. First, the diagnosis of stroke was based on self-reported physician diagnosis, without review of hospital records or imaging confirmation, which may introduce misclassification bias. Although linkage with hospital records would remarkably improve the accuracy of outcome ascertainment, this was not feasible for the current CHARLS dataset due to the legal and administrative barriers to linking survey data with national hospital databases in China. However, the use of self-reported physician-diagnosed stroke has been widely employed and validated in other major international aging studies, which were similar to CHARLS, such as the Health and Retirement Study and the English Longitudinal Study of Aging, supporting its utility as a proxy in epidemiological research. Additionally, atherosclerosis is a primary pathological basis of stroke development; however, our study lacked imaging biomarkers of atherosclerosis (e.g., carotid ultrasound). Future studies incorporating these imaging biomarkers are warranted to confirm our findings and explore the underlying mechanisms more directly. Second, our analysis was limited by the lack of data on stroke subtypes. Stroke is pathophysiologically categorized into two primary types: ischemic and hemorrhagic. Ischemic stroke, constituting the majority of cases, results from cerebral vessel occlusion and can be further subdivided into subtypes such as extensive atherosclerosis of the arteries, cardioembolic infarction, lacunar infarction, and others. Hemorrhagic stroke arises from vessel rupture and is primarily classified as intracerebral hemorrhage or subarachnoid hemorrhage [37]. Due to the inability to distinguish subtypes, we cannot determine whether the association between TyG-CI and stroke risk is consistent or differential across subtypes. Third, although we adjusted for potential confounders, unmeasured variables might residually influence outcomes. Fourth, drug intervention could not be rigorously analyzed due to incomplete longitudinal medication adherence records across follow-up, a common challenge in observational cohort designs. Finally, the focus on adults aged ≥45 years may restrict generalizability to younger populations, where emerging metabolic disorders are increasingly linked to early-onset cerebrovascular events.

## 5. Conclusions

In conclusion, our study demonstrates that elevated TyG, CI, and the novel composite TyG-CI are independently associated with increased stroke risk. Notably, TyG-CI exhibited higher predictive accuracy for incident stroke compared to its individual components, positioning it as an effective stratification tool for stroke risk evaluation in clinical practice. Further research is essential to validate the clinical applicability of TyG-CI in various clinical settings.

## Figures and Tables

**Table 1:** Baseline characteristics of participants according to the quartile of TyG-CI.

Characteristic	Overall *n* = 8011	Q1 *n* = 2003	Q2 *n* = 2003	Q3 *n* = 2003	Q4 *n* = 2002	*p* Value
Age, years	58.0 (52.0, 65.0)	56.0 (49.0, 62.0)	58.0 (51.0, 65.0)	59.0 (53.0, 65.0)	60.0 (54.0, 67.0)	<0.001
Sex, %						<0.001
Male	3701 (46%)	1117 (56%)	1038 (52%)	834 (42%)	712 (36%)	
Female	4310 (54%)	886 (44%)	965 (48%)	1169 (58%)	1290 (64%)	
Residence, %						<0.001
Rural	6658 (83%)	1729 (86%)	1695 (85%)	1653 (83%)	1581 (79%)	
City	1353 (17%)	274 (14%)	308 (15%)	350 (17%)	421 (21%)	
Marital status, %						<0.001
Non-married	982 (12%)	227 (11%)	229 (11%)	227 (11%)	299 (15%)	
Married	7029 (88%)	1776 (89%)	1774 (89%)	1776 (89%)	1703 (85%)	
Education level						0.70
High school or below	7764 (97%)	1941 (97%)	1946 (97%)	1944 (97%)	1933 (97%)	
College or above	247 (3.1%)	62 (3.1%)	57 (2.8%)	59 (2.9%)	69 (3.4%)	
Smoking, %	3105 (39%)	917 (46%)	839 (42%)	691 (34%)	658 (33%)	<0.001
Drinking, %	3132 (39%)	882 (44%)	848 (42%)	721 (36%)	681 (34%)	<0.001
Hypertension, %	3255 (41%)	515 (26%)	698 (35%)	878 (44%)	1164 (58%)	<0.001
Dyslipidemia, %	3834 (48%)	423 (21%)	713 (36%)	1074 (54%)	1624 (81%)	<0.001
Diabetes, %	713 (8.9%)	64 (3.2%)	69 (3.4%)	163 (8.1%)	417 (21%)	<0.001
Heart disease, %	928 (12%)	161 (8.0%)	203 (10%)	257 (13%)	307 (15%)	<0.001
Kidney disease, %	702 (8.8%)	181 (9.0%)	165 (8.2%)	177 (8.8%)	179 (8.9%)	0.80
BMI, kg/m^2^	23.1 (20.9, 25.8)	21.6 (19.7, 23.5)	22.4 (20.4, 24.6)	24.0 (21.6, 26.3)	25.1 (22.7, 27.8)	<0.001
SBP, mmHg	126.0 (113.5, 141.0)	120.0 (109.5, 133.5)	124.0 (112.5, 138.5)	128.0 (115.5, 142.5)	133.0 (120.0, 147.5)	<0.001
DBP, mmHg	74.5 (66.5, 82.5)	71.5 (64.5, 80.0)	73.5 (66.0, 81.5)	75.0 (67.5, 83.5)	77.5 (70.0, 85.5)	<0.001
TC, mg/dL	191.4 (168.6, 216.5)	179.0 (159.3, 202.2)	188.7 (165.5, 211.1)	195.6 (173.2, 219.6)	204.7 (180.5, 233.1)	<0.001
TG, mg/dL	104.4 (74.3, 150.4)	67.3 (54.0, 85.0)	91.2 (72.6, 114.2)	115.9 (92.9, 149.6)	180.5 (133.6, 257.5)	<0.001
HDL-C, mg/dL	49.5 (40.6, 60.3)	57.2 (48.7, 67.7)	52.2 (43.7, 62.6)	47.9 (40.6, 57.6)	41.4 (34.0, 49.1)	<0.001
LDL-C, mg/dL	115.2 (94.3, 138.4)	107.5 (90.1, 127.6)	115.6 (95.5, 136.5)	121.4 (100.5, 143.8)	117.9 (92.8, 144.2)	<0.001
FPG, mg/dL	102.2 (94.5, 112.5)	96.7 (90.4, 104.4)	99.9 (93.4, 107.6)	103.1 (95.9, 112.7)	112.0 (101.9, 134.6)	<0.001
HbA1c, %	5.2 (4.9, 5.4)	5.1 (4.8, 5.3)	5.1 (4.8, 5.4)	5.2 (4.9, 5.5)	5.3 (5.0, 5.8)	<0.001
eGFR, mL/min/1.73 m^2^	98.9 (88.6, 105.6)	101.3 (92.4, 107.7)	99.5 (90.1, 105.9)	98.1 (87.5, 104.7)	96.1 (84.3, 103.7)	<0.001
TyG	8.6 (8.2, 9.0)	8.1 (7.9, 8.3)	8.4 (8.2, 8.7)	8.7 (8.5, 9.0)	9.3 (8.9, 9.7)	<0.001
CI	1.3 (1.2, 1.3)	1.2 (1.2, 1.2)	1.3 (1.2, 1.3)	1.3 (1.3, 1.3)	1.4 (1.3, 1.4)	<0.001
TyG-CI	11.0 (10.2, 11.9)	9.7 (9.3, 10.0)	10.7 (10.5, 10.9)	11.4 (11.2, 11.7)	12.6 (12.2, 13.2)	<0.001

Data were presented as median (Quartile 1, Quartile 3) for continuous measures, and *n* (%) for categorical measures. Abbreviations: Q, quartile; BMI, body mass index; SBP, systolic blood pressure; DBP, diastolic blood pressure; TC, total cholesterol; TG, triglyceride; HDL-C, high-density lipoprotein cholesterol; LDL-C, low-density lipoprotein cholesterol; FPG, fasting plasma glucose; HbA1c, glycated hemoglobin; eGFR, estimated glomerular filtration ratio; TyG, triglyceride–glucose; CI, conicity index; TyG-CI, triglyceride glucose–conicity index.

**Table 2:** Association of TyG, CI, and TyG-CI with stroke risk.

	Hazard Ratio (95%CI)		
	Model 1	Model 2	Model 3
TyG quartile			
Q1	1.00 (Reference)	1.00 (Reference)	1.00 (Reference)
Q2	1.48 (1.18–1.87)	1.51 (1.20–1.91)	1.38 (1.09–1.74)
Q3	1.97 (1.58–2.45)	1.99 (1.59–2.49)	1.60 (1.27–2.02)
Q4	2.00 (1.61–2.50)	2.08 (1.66–2.60)	1.36 (1.05–1.76)
TyG Per IQR	1.31 (1.21–1.41)	1.33 (1.23–1.43)	1.12 (1.01–1.23)
CI quartile			
Q1	1.00 (Reference)	1.00 (Reference)	1.00 (Reference)
Q2	1.09 (0.86–1.36)	1.06 (0.84–1.34)	1.01 (0.80–1.27)
Q3	1.43 (1.15–1.77)	1.39 (1.12–1.72)	1.16 (0.93–1.44)
Q4	1.97 (1.61–2.42)	1.81 (1.46–2.24)	1.43 (1.15–1.78)
CI Per IQR	1.12 (1.07–1.17)	1.09 (1.04–1.15)	1.06 (1.00–1.13)
TyG-CI quartile			
Q1	1.00 (Reference)	1.00 (Reference)	1.00 (Reference)
Q2	1.47 (1.16–1.87)	1.43 (1.13–1.82)	1.31 (1.03–1.66)
Q3	1.85 (1.47–2.33)	1.83 (1.45–2.30)	1.48 (1.16–1.88)
Q4	2.51 (2.01–3.12)	2.42 (1.93–3.03)	1.69 (1.31–2.17)
TyG-CI Per IQR	1.26 (1.19–1.33)	1.23 (1.16–1.31)	1.11 (1.03–1.21)

Model 1: not adjust for other covariates. Model 2: adjust for age, sex, residence, marital status, education level, smoking status, and drinking status. Model 3: adjust for age, sex, residence, marital status, education level, smoking status, drinking status, body mass index, hypertension, dyslipidemia, diabetes, heart disease, and kidney disease. Abbreviations: TyG, triglyceride–glucose; CI, conicity index; TyG-CI, triglyceride glucose–conicity index; Q, quartile; 95%CI, 95% confidence interval; IQR, interquartile range.

## Data Availability

The data supporting the findings of this study are available on the CHARLS website (http://charls.pku.edu.cn/), accessed on 25 April 2025.

## Supplementary Materials

The following supporting information can be downloaded at: https://www.mdpi.com/article/10.3390/jcm14197086/s1, Table S1: STROBE Checklist of items that should be included in reports of observational studies; Table S2: Baseline characteristics of participants according to stroke status; Table S3: Data comparison before and after imputation of missing covariates; Table S4: Subgroup analysis for the association of TyG-CI and stroke risk; Table S5: Association between TyG-CI and stroke risk: sensitivity analyses; Table S6: Baseline characteristics of participants with low TyG-CI values (Q1) according to stroke status; Table S7: Association between risk factors and incident stroke in participants with low TyG-CI values (Q1).

## Abbreviations

The following abbreviations are used in this manuscript:

AO	Abdominal obesity
AUC	Area under the curve
BMI	Body mass index
CHARLS	China Health and Retirement Longitudinal Study
CHD	Coronary heart disease
CI	Conicity index
CVD	Cardiovascular disease
DBP	Diastolic blood pressure
eGFR	Estimated glomerular filtration rate
FPG	Fasting plasma glucose
HbA1c	Glycosylated hemoglobin A1c
HDL-C	High-density lipoprotein cholesterol
HOMA-IR	Homeostatic model assessment for insulin resistance
HR	Hazard ratio
IR	Insulin resistance
LDL-C	Low-density lipoprotein cholesterol
OR	Odds ratio
Q	Quartile
RCS	Restricted cubic spline
ROC	Receiver operating characteristic curve
SBP	Systolic blood pressure
TC	Total cholesterol
TG	Triglycerides
TyG	Triglyceride–glucose index
TyG-CI	Triglyceride glucose–conicity index
WC	Waist circumference
95%CI	95% confidence interval
