# Supplementary material for: The Triglyceride Glucose–Conicity Index as a Novel Predictor for Stroke Risk: A Nationwide Prospective Cohort Study
Source: J Clin Med. 2025 Oct 7;14(19):7086. doi: 10.3390/jcm14197086 (PMC12524681; doi:10.3390/jcm14197086)
Supplement: Supplementary file 1 [file jcm-14-07086-s001.zip › jcm-3809495-supplementary.pdf]

Supplementary Materials

Table S1. STROBE Checklist of items that should be included in reports of observational studies. 2
Table S2. Baseline characteristics of participants according to stroke status........ 4
Table S3. Data comparison before and after imputation of missing covariates. 6
Table S4. Subgroup analysis for the association of TyG-CI and stroke risk. 7
Table S5. Association between TyG-CI and stroke risk: sensitivity analyses. 8
Table S6. Baseline characteristics of participants with low TyG-CI values (Q1) according to stroke status. 9
Table S7. Association between risk factors and incident stroke in participants with low TyG-CI values (Q1). 10

Table S1. STROBE Checklist of items that should be included in reports of cohort studies.

	Item No	Recommendation	Page No.
**Title and abstract**	1	(a) Indicate the study's design with a commonly used term in the title or the abstract	1
		(b) Provide in the abstract an informative and balanced summary of what was done and what was found	1
**Introduction**			
Background/rationale	2	Explain the scientific background and rationale for the investigation being reported	1,2
Objectives	3	State specific objectives, including any prespecified hypotheses	2
**Methods**			
Study design	4	Present key elements of study design early in the paper	2
Setting	5	Describe the setting, locations, and relevant dates, including periods of recruitment, exposure, follow-up, and data collection	2
Participants	6	(a) Give the eligibility criteria, and the sources and methods of selection of participants. Describe methods of follow-up	2,3
		(b) For matched studies, give matching criteria and number of exposed and unexposed	NA
Variables	7	Clearly define all outcomes, exposures, predictors, potential confounders, and effect modifiers. Give diagnostic criteria, if applicable	3,4
Data sources/ measurement	8*	For each variable of interest, give sources of data and details of methods of assessment (measurement). Describe comparability of assessment methods if there is more than one group	3,4
Bias	9	Describe any efforts to address potential sources of bias	5
Study size	10	Explain how the study size was arrived at	2,3
Quantitative variables	11	Explain how quantitative variables were handled in the analyses. If applicable, describe which groupings were chosen and why	4
Statistical methods	12	(a) Describe all statistical methods, including those used to control for confounding	4,5
		(b) Describe any methods used to examine subgroups and interactions	5
		(c) Explain how missing data were addressed	4,5
		(d) If applicable, explain how loss to follow-up was addressed	NA
		(e) Describe any sensitivity analyses	5

**Results**			
Participants	13*	(a) Report numbers of individuals at each stage of study—eg numbers potentially eligible, examined for eligibility, confirmed eligible, included in the study, completing follow-up, and analysed	2,3,5
		(b) Give reasons for non-participation at each stage	NA
		(c) Consider use of a flow diagram	Figure 1
Descriptive data	14*	(a) Give characteristics of study participants (eg demographic, clinical, social) and information on exposures and potential confounders	5,6
		(b) Indicate number of participants with missing data for each variable of interest	Figure 1
		(c) Summarise follow-up time (eg, average and total amount)	6
Outcome data	15*	Report numbers of outcome events or summary measures over time	6
Main results	16	(a) Give unadjusted estimates and, if applicable, confounder-adjusted estimates and their precision (eg, 95% confidence interval). Make clear which confounders were adjusted for and why they were included	6,7
		(b) Report category boundaries when continuous variables were categorized	6
		(c) If relevant, consider translating estimates of relative risk into absolute risk for a meaningful time period	NA
Other analyses	17	Report other analyses done—eg analyses of subgroups and interactions, and sensitivity analyses	8
**Discussion**			
Key results	18	Summarise key results with reference to study objectives	8-11
Limitations	19	Discuss limitations of the study, taking into account sources of potential bias or imprecision. Discuss both direction and magnitude of any potential bias	10
Interpretation	20	Give a cautious overall interpretation of results considering objectives, limitations, multiplicity of analyses, results from similar studies, and other relevant evidence	8-11
Generalisability	21	Discuss the generalisability (external validity) of the study results	10
**Other information**			
Funding	22	Give the source of funding and the role of the funders for the present study and, if applicable, for the original study on which the present article is based	11

*Give information separately for exposed and unexposed groups.

Note: An Explanation and Elaboration article discusses each checklist item and gives methodological background and published examples of transparent reporting. The STROBE checklist is best used in conjunction with this article (freely available on the Web sites of PLoS Medicine at http://www.plosmedicine.org/, Annals of Internal Medicine at http://www.annals.org/, and Epidemiology at http://www.epidem.com/). Informat ion on the STROBE Initiative is available at http://www.strobe-statement.org.

Table S2. Baseline characteristics of participants according to stroke status.

Characteristic	Overall<br>N = 8011	No stroke<br>N =7258	Stroke<br>N =753	P value
Age, years	58.0 (52.0, 65.0)	58.0 (52.0, 65.0)	60.0 (55.0, 67.0)	<0.001
Sex, %				0.30
Male	3,701 (46%)	3,339 (46%)	362 (48%)	
Female	4,310 (54%)	3,919 (54%)	391 (52%)	
Residence, %				0.90
Rural	6,658 (83%)	6,034 (83%)	624 (83%)	
City	1,353 (17%)	1,224 (17%)	129 (17%)	
Marital status, %				0.086
Non-married	982 (12%)	875 (12%)	107 (14%)	
Married	7,029 (88%)	6,383 (88%)	646 (86%)	
Education level				0.50
High school or below	7,764 (97%)	7,031 (97%)	733 (97%)	
College or above	247 (3.1%)	227 (3.1%)	20 (2.7%)	
Smoking, %	3,105 (39%)	2,786 (38%)	319 (42%)	0.033
Drinking, %	3,132 (39%)	2,811 (39%)	321 (43%)	0.037
Hypertension, %	3,255 (41%)	2,799 (39%)	456 (61%)	<0.001
Dyslipidemia, %	3,834 (48%)	3,393 (47%)	441 (59%)	<0.001
Diabetes, %	713 (8.9%)	616 (8.5%)	97 (13%)	<0.001
Heart disease, %	928 (12%)	781 (11%)	147 (20%)	<0.001
Kidney disease, %	702 (8.8%)	616 (8.5%)	86 (11%)	0.007
BMI, kg/m^2	23.1 (20.9, 25.8)	23.1 (20.8, 25.7)	24.1 (21.7, 26.9)	<0.001
SBP, mmHg	126.0 (113.5, 141.0)	125.5 (113.5, 140.0)	134.0 (119.5, 150.0)	<0.001
DBP, mmHg	74.5 (66.5, 82.5)	74.0 (66.5, 82.5)	78.0 (70.0, 87.0)	<0.001
TC, mg/dL	191.4 (168.6, 216.5)	191.0 (168.2, 216.1)	195.2 (171.3, 220.4)	0.005
TG, mg/dL	104.4 (74.3, 150.4)	102.7 (73.5, 149.6)	115.1 (85.0, 165.5)	<0.001
HDL-C, mg/dL	49.5 (40.6, 60.3)	49.9 (41.0, 60.7)	47.2 (39.4, 56.4)	<0.001
LDL-C, mg/dL	115.2 (94.3, 138.4)	114.8 (94.3, 137.6)	119.5 (98.2, 141.9)	0.001
FPG, mg/dL	102.2 (94.5, 112.5)	102.1 (94.5, 112.1)	104.4 (95.9, 116.8)	<0.001
HbA1c, %	5.2 (4.9, 5.4)	5.1 (4.9, 5.4)	5.2 (4.9, 5.6)	<0.001
eGFR, mL/min/1.73m^2	98.9 (88.6, 105.6)	99.1 (88.9, 105.8)	96.9 (86.2, 103.2)	<0.001
TyG	8.6 (8.2, 9.0)	8.6 (8.2, 9.0)	8.7 (8.4, 9.1)	<0.001
CI	1.3 (1.2, 1.3)	1.3 (1.2, 1.3)	1.3 (1.2, 1.4)	<0.001
TyG-CI	11.0 (10.2, 11.9)	11.0 (10.2, 11.9)	11.4 (10.6, 12.3)	<0.001

Data were presented as median (Quartile 1, Quartile 3) for continuous measures, and n (%) for categorical measures.

Abbreviations: BMI, body mass index; SBP, systolic blood pressure; DBP, diastolic blood pressure; TC, total cholesterol; TG, triglyceride; HDL-C, high-density lipoprotein cholesterol; LDL-C, low-density lipoprotein cholesterol; FPG, fasting plasma glucose; HbA1c, glycated hemoglobin; eGFR, estimated glomerular filtration ratio; TyG, triglyceride-glucose; CI, conicity index; TyG-CI, triglyceride glucose-conicity index.

Table S3. Data comparison before and after imputation of missing covariates.

Characteristic	Impute data<br>N = 8011	Complete data<br>N = 7714	P value
Age, years	58.0 (52.0, 65.0)	58.0 (52.0, 65.0)	>0.9
Sex, %			0.9
Male	3,701 (46%)	3,573 (46%)	
Female	4,310 (54%)	4,141 (54%)	
Residence, %			0.9
Rural	6,658 (83%)	6,404 (83%)	
City	1,353 (17%)	1,310 (17%)	
Marital status, %			0.7
Non-married	982 (12%)	930 (12%)	
Married	7,029 (88%)	6,784 (88%)	
Education level			>0.9
High school or below	7,764 (97%)	7,475 (97%)	
College or above	247 (3.1%)	239 (3.1%)	
Smoking, %	3,105 (39%)	3,003 (39%)	0.8
Drinking, %	3,132 (39%)	3,026 (39%)	0.9
Hypertension, %	3,255 (41%)	3,159 (41%)	0.7
Dyslipidemia, %	3,834 (48%)	3,719 (48%)	0.7
Diabetes, %	713 (8.9%)	697 (9.0%)	0.8
Heart disease, %	928 (12%)	911 (12%)	0.7
Kidney disease, %	702 (8.8%)	675 (8.8%)	>0.9
BMI, kg/m^2	23.1 (20.9, 25.8)	23.2 (20.9, 25.8)	>0.9
TyG	8.6 (8.2, 9.0)	8.6 (8.2, 9.0)	0.8
CI	1.3 (1.2, 1.3)	1.3 (1.2, 1.3)	>0.9
TyG-CI	11.0 (10.2, 11.9)	11.0 (10.3, 11.9)	0.8

Data were presented as median (Quartile 1, Quartile 3) for continuous measures, and n (%) for categorical measures.

Abbreviations: BMI, body mass index; TyG, triglyceride-glucose; CI, conicity index; TyG-CI, triglyceride glucose-conicity index.

Table S4. Subgroup analysis for the association of TyG-CI and stroke risk.

Subgroup	N	Hazard ratio (95%CI)	P for interaction
Age			0.31
<60 years	4495	1.16 (1.02-1.32)	
≥60 years	3516	1.08 (0.96-1.21)	
Sex			0.44
Male	3701	1.10 (0.97-1.25)	
Female	4310	1.13 (1.01-1.25)	
Smoking			0.41
No	4906	1.10 (1.00-1.23)	
Yes	3105	1.12 (0.98-1.29)	
Drinking			0.69
No	4879	1.15 (1.04-1.28)	
Yes	3132	1.08 (0.94-1.22)	
Hypertension			0.16
No	4756	1.16 (1.05-1.30)	
Yes	3255	1.07 (0.96-1.19)	
Dyslipidemia			0.39
No	4177	1.16 (1.02-1.31)	
Yes	3834	1.09 (0.98-1.21)	
Diabetes			0.82
No	7298	1.12 (1.02-1.22)	
Yes	713	1.11 (0.90-1.37)	
Heart disease			0.23
No	7083	1.12 (1.02-1.22)	
Yes	928	1.08 (0.91-1.29)	
Kidney disease			0.56
No	7309	1.12 (1.02-1.22)	
Yes	702	1.10 (0.86-1.40)	

All hazard ratios were expressed as per interquartile range increase of TyG-CI and were adjust for age, sex, residence, marital status, education level, smoking status, drinking status, body mass index, hypertension, dyslipidemia, diabetes, heart disease, and kidney disease.

Abbreviations: TyG-CI, triglyceride glucose-conicity index; 95%CI, 95% confidence interval.

Table S5. Association between TyG-CI and stroke risk: sensitivity analyses.

	Hazard ratio (95%CI)		
	**Model 1**	**Model 2**	**Model 3**
**Excluding participants receiving antihyperglycemic treatment, antihypertensive treatment, or lipid-lowering treatment**			
**TyG-CI quartile**			
Q1	1.00 (Reference)	1.00 (Reference)	1.00 (Reference)
Q2	1.49 (1.10-2.02)	1.44 (1.06-1.95)	1.38 (1.02-1.87)
Q3	2.14 (1.61-2.85)	2.09 (1.57-2.79)	1.86 (1.38-2.50)
Q4	2.17 (1.63-2.88)	2.10 (1.57-2.81)	1.67 (1.22-2.30)
**TyG-CI Per IQR**	1.21 (1.13-1.30)	1.19 (1.11-1.29)	1.13 (1.02-1.24)
**Complete case analysis**			
**TyG-CI quartile**			
Q1	1.00 (Reference)	1.00 (Reference)	1.00 (Reference)
Q2	1.50 (1.18-1.91)	1.46 (1.15-1.86)	1.33 (1.04-1.69)
Q3	1.87 (1.48-2.35)	1.84 (1.46-2.33)	1.48 (1.16-1.88)
Q4	2.45 (1.97-3.06)	2.36 (1.88-2.97)	1.63 (1.26-2.10)
**TyG-CI Per IQR**	1.25 (1.18-1.32)	1.22 (1.15-1.30)	1.10 (1.01-1.20)

Model 1: not adjust for other covariates

Model 2: adjust for age, sex, residence, marital status, education level, smoking status, and drinking status.

Model 3: adjust for age, sex, residence, marital status, education level, smoking status, drinking status, body mass index, hypertension, dyslipidemia, diabetes, heart disease, and kidney disease.

Abbreviations: TyG-CI, triglyceride glucose-conicity index; 95%CI, 95% confidence interval; IQR, interquartile range.

Table S6. Baseline characteristics of participants with low TyG-CI values (Q1) according to stroke status.

Characteristic	Overall<br>N = 2003	No stroke<br>N =1888	Stroke<br>N =115	P value
Age, years	56.0 (49.0, 62.0)	56.0 (49.0, 62.0)	58.0 (52.0, 67.0)	0.014
Sex, %				0.90
Male	1,117 (56%)	1,052 (56%)	65 (57%)	
Female	886 (44%)	836 (44%)	50 (43%)	
Residence, %				0.40
Rural	1,729 (86%)	1,627 (86%)	102 (89%)	
City		261 (14%)	13 (11%)	
Marital status, %				0.13
Non-married	227 (11%)	209 (11%)	18 (16%)	
Married	1,776 (89%)	1,679 (89%)	97 (84%)	
Education level				0.60
High school or below	1,941 (97%)	1,828 (97%)	113 (98%)	
College or above	62 (3.1%)	60 (3.2%)	2 (1.7%)	
Smoking, %	917 (46%)	861 (46%)	56 (49%)	0.50
Drinking, %	882 (44%)	826 (44%)	56 (49%)	0.30
Hypertension, %	515 (26%)	456 (24%)	59 (51%)	<0.001
Dyslipidemia, %	423 (21%)	387 (20%)	36 (31%)	0.006
Diabetes, %	64 (3.2%)	58 (3.1%)	6 (5.2%)	0.30
Heart disease, %	161 (8.0%)	137 (7.3%)	24 (21%)	<0.001
Kidney disease, %	181 (9.0%)	164 (8.7%)	17 (15%)	0.027
BMI, kg/m^2	21.6 (19.7, 23.5)	21.5 (19.7, 23.4)	22.2 (20.4, 24.0)	0.033
SBP, mmHg	120.0 (109.5, 133.5)	119.5 (109.3, 132.5)	130.5 (115.0, 149.0)	<0.001
DBP, mmHg	71.5 (64.5, 80.0)	71.5 (64.0, 79.5)	77.5 (69.0, 86.5)	<0.001
TC, mg/dL	179.0 (159.3, 202.2)	179.4 (159.7, 202.2)	174.4 (154.6, 197.6)	0.20
TG, mg/dL	67.3 (54.0, 85.0)	67.3 (54.0, 85.0)	71.7 (60.2, 92.0)	0.009
HDL-C, mg/dL	57.2 (48.7, 67.7)	57.2 (48.9, 68.0)	54.9 (46.0, 63.8)	0.015
LDL-C, mg/dL	107.5 (90.1, 127.6)	107.9 (90.3, 127.6)	102.4 (87.8, 122.9)	0.20
FPG, mg/dL	96.7 (90.4, 104.4)	96.7 (90.0, 104.4)	96.1 (92.5, 104.8)	0.20
HbA1c, %	5.1 (4.8, 5.3)	5.1 (4.8, 5.3)	5.1 (4.8, 5.4)	0.14
eGFR, $mL/min/1.73m^2$	101.3 (92.4, 107.7)	101.3 (92.6, 107.8)	101.4 (89.8, 107.3)	0.60

Data were presented as median (Quartile 1, Quartile 3) for continuous measures, and n (%) for categorical measures.

Abbreviations: BMI, body mass index; SBP, systolic blood pressure; DBP, diastolic blood pressure; TC, total cholesterol; TG, triglyceride; HDL-C, high-density lipoprotein cholesterol; LDL-C, low-density lipoprotein cholesterol; FPG, fasting plasma glucose; HbA1c, glycated hemoglobin; eGFR, estimated glomerular filtration ratio.

Table S7. Association between risk factors and incident stroke in participants with low TyG-CI values (Q1).

	Univariate analysis		Multivariate analysis	
**Risk factors**	**Hazard ratio (95%CI)**	**P value**	**Hazard ratio (95%CI)**	**P value**
Age	1.28 (1.07-1.52)	0.006	1.14 (0.94-1.40)	0.18
Female	0.95 (0.66-1.38)	0.80	-	-
City	0.85 (0.47-1.51)	0.57	-	-
Married	0.65 (0.40-1.08)	0.099	-	-
College or above	0.57 (0.14-2.29)	0.42	-	-
Smoking	1.14 (0.79-1.65)	0.48	-	-
Drinking	1.23 (0.85-1.77)	0.27	-	-
Hypertension	3.33 (2.31-4.80)	<0.001	1.72 (1.01-2.90)	0.045
Dyslipidemia	1.74 (1.17-2.58)	0.006	1.12 (0.71-1.77)	0.61
Diabetes	1.66 (0.73-3.78)	0.23	-	-
Heart disease	3.37 (2.15-5.28)	<0.001	2.58 (1.62-4.11)	<0.001
Kidney disease	1.80 (1.07-3.01)	0.026	1.37 (0.81-2.32)	0.25
BMI	0.98 (0.75-1.27)	0.87	-	-
SBP	1.63 (1.40-1.89)	<0.001	1.25 (0.93-1.69)	0.14
DBP	1.54 (1.31-1.82)	<0.001	1.11 (0.84-1.47)	0.48
TC	0.94 (0.78-1.13)	0.50	-	-
TG	1.26 (1.15-1.37)	<0.001	1.19 (1.05-1.34)	0.006
HDL-C	0.81 (0.66-0.98)	0.032	0.88 (0.71-1.09)	0.23
LDL-C	0.88 (0.73-1.07)	0.21	-	-
FPG	1.10 (0.95-1.26)	0.21	-	-
HbA1c	1.07 (0.92-1.25)	0.38	-	-
eGFR	0.92 (0.77-1.09)	0.34	-	-

Variables with a P value < 0.05 in the univariate Cox analysis were included in the multivariate model. Hazard ratios are presented per 1-standard deviation increase for continuous variables and for the presence vs. absence of a condition for categorical variables.

Abbreviations: BMI, body mass index; SBP, systolic blood pressure; DBP, diastolic blood pressure; TC, total cholesterol; TG, triglyceride; HDL-C, high-density lipoprotein cholesterol; LDL-C, low-density lipoprotein cholesterol; FPG, fasting plasma glucose; HbA1c, glycated hemoglobin; eGFR, estimated glomerular filtration ratio.
